# Metabarcoding of fungal communities associated with bark beetles

**DOI:** 10.1002/ece3.1925

**Published:** 2016-02-12

**Authors:** Kirsten E. Miller, Kevin Hopkins, Daegan J. G. Inward, Alfried P. Vogler

**Affiliations:** ^1^ Department of Life SciencesNatural History MuseumCromwell RoadLondonSW7 5BDUK; ^2^ Department of Life SciencesImperial College LondonSilwood Park CampusAscotSL5 7PYUK; ^3^ Centre for Ecosystems, Society and BiosecurityForest ResearchAlice Holt LodgeFarnhamSurreyGU10 4LHUK

**Keywords:** Illumina, ITS2, Ophiostomaceae, primer tags, Scolytinae, tree pathogens

## Abstract

Many species of fungi are closely allied with bark beetles, including many tree pathogens, but their species richness and patterns of distribution remain largely unknown. We established a protocol for metabarcoding of fungal communities directly from total genomic DNA extracted from individual beetles, showing that the ITS3/4 primer pair selectively amplifies the fungal ITS. Using three specimens of bark beetle from different species, we assess the fungal diversity associated with these specimens and the repeatability of these estimates in PCRs conducted with different primer tags. The combined replicates produced 727 fungal Operational Taxonomic Units (OTUs) for the specimen of *Hylastes ater*, 435 OTUs for *Tomicus piniperda*, and 294 OTUs for *Trypodendron lineatum,* while individual PCR reactions produced on average only 229, 54, and 31 OTUs for the three specimens, respectively. Yet, communities from PCR replicates were very similar in pairwise comparisons, in particular when considering species abundance, but differed greatly among the three beetle specimens. Different primer tags or the inclusion of amplicons in separate libraries did not impact the species composition. The ITS2 sequences were identified with the Lowest Common Ancestor approach and correspond to diverse lineages of fungi, including Ophiostomaceae and Leotiomycetes widely found to be tree pathogens. We conclude that Illumina MiSeq metabarcoding reliably captures fungal diversity associated with bark beetles, although numerous PCR replicates are recommended for an exhaustive sample. Direct PCR from beetle DNA extractions provides a rapid method for future surveys of fungal species diversity and their associations with bark beetles and environmental variables.

## Introduction

Bark beetles (Scolytinae) are among the most destructive of forest pests, but much of their impact on the ecosystem is due to the associations they form with fungi (Paine et al. 1997). Numerous fungal taxa have been isolated from these beetles and their galleries, including phytopathogenic species, and many of these associations represent symbiotic relationships (Six 2004). Fungal associates often provide important nutritional benefits to the beetle, and ambrosia beetles, which transport and cultivate symbiotic fungi as their primary food source, notably form a high proportion of the invasive scolytine species to have successfully established in North America (Haack 2006) and Europe (Kirkendall and Faccoli 2010). Phytopathogenic fungi may assist in overwhelming the defences of a host tree and facilitate a successful attack by the beetle (Paine et al. 1997), but pathogenicity may also provide the fungal associates with their own competitive advantage within the host tree (Six and Wingfield 2011). Current knowledge of this system is biased toward a relatively small subset of economically important species. However, reports are accruing of unforeseen levels of damage caused by both native and invasive bark beetle species across the globe (Humble and Allen 2006; Hulcr and Dunn 2011). The causes of these occurrences are not well understood, but there is evidence to suggest they result from a combination of environmental change, active and passive movement of beetle hosts and fungal associates, and changes in pathogenicity (Hulcr and Dunn 2011; Ploetz et al. 2013; Six 2013). It is clear that we need to take a more holistic approach toward understanding these interactions in order to better predict how environmental change might affect a native community and also how invasive species might integrate into a new ecosystem.

Recent developments in high‐throughput sequencing (HTS) have revolutionized the study of fungal communities, revealing unexpectedly high diversity that greatly surpasses counts from morphological studies and culturing (Hawksworth 2012; Tedersoo et al. 2014). The great sequencing depth achievable with recent HTS methods permits the characterization of entire microbial communities comprising hundreds of species that are typically found in a single environmental sample (Taylor et al. 2008; Schmidt et al. 2013; Tedersoo et al. 2014). A convenient genome region for studying this diversity is the Internal Transcribed Spacer (ITS) of the rRNA locus that has emerged as a standard ‘barcode’ marker for fungal species detection and delimitation (Schoch et al. 2012) Using this marker, the amplification from mixed samples (‘metabarcoding’) provides new possibilities for studying the interactions of fungi and bark beetles, but to date only a single study has integrated HTS and bark beetle associated fungal communities. Using 454 pyrosequencing, Kostovcik et al. (2015) investigated the mycobiota of three ambrosia beetle species, with focus on the fungal communities isolated from the mycangia, that is, the specialized structures of the exoskeleton in many bark beetles designed for the transport of fungal spores. They found several dozen fungal Operational Taxonomic Units (OTUs) associated with the mycangia of every specimen, which represents many more species than could be isolated with traditional culturing techniques. Additionally, the three beetle species included in this study displayed divergent mycobiota, and diversity was further increased by geographic variation. These findings suggest that the fungal diversity in this system is indeed very large and that it cannot be sufficiently captured by conventional methods.

While the study by Kostovcik et al. (2015) focuses on the mycangia of ambrosia beetles, it is known that the spores and mycelia of pathogenic and symbiotic fungi can be transported either affixed to beetle cuticles externally or internally within the insect gut (Harrington 2005). The full mycobiome of a beetle may be captured if DNA is extracted from the entire beetle specimen. Fungal sequences can then be obtained by PCR amplification of ITS with fungus‐specific primers that are believed to discriminate between beetle and fungal DNA templates (De Beeck et al. 2014). This approach also circumvents the need for tedious preparation of the mycangia and thus permits the rapid analysis of numerous samples. High sample numbers raise the prospect of establishing patterns that link fungal taxa with beetle (host) species or environmental conditions through frequency of association (Wright and Reeves 1992).

Processing of large numbers of specimens with HTS approaches also requires an efficient system for separation of these samples in the sequencing process. The addition of a sequence ‘tag’ at the amplification step is commonly used to attach a sample‐specific nucleotide identifier to each primer, so that resulting amplicons are separated in the postsequencing bioinformatics analysis (Gloor et al. 2010). However, adding different tags to the primers may subtly change the outcome of the PCR amplification and thus unique tags may cause tag‐specific biases in community composition, as has been observed in bacterial communities (Berry et al. 2011; Faircloth and Glenn 2012).

The current study tests the feasibility of a HTS approach to the metabarcoding of fungal communities associated with bark beetles. First, we address the concern that fungus‐specific ITS primers are not sufficiently taxon specific to discriminate against the much greater amount of beetle template in the DNA extract. Secondly, we test the reproducibility of individual PCRs in regard to species composition and abundance, in particular, as differently tagged primers are used that may amplify slightly different sets of fungal template from the mixtures. The tests were based on 15 newly designed primer tags for the ITS3/4 primer pair widely used for amplification of the fungal ITS2 regions, followed by Illumina MiSeq sequencing of the resulting amplicons. We thus ask if metabarcoding of fungal communities can be achieved by amplicon sequencing directly from total DNA extracted from the insects, and how reproducible the recovery of complex fungal communities is when using differently tagged primers. The latter was addressed by replicated PCRs on a test set of thee DNA extractions from different beetle species and counts of fungal species and assessment of the fungal communities.

## Materials and Methods

### Samples used and experimental design

One specimen each of *Hylastes ater, Tomicus piniperda*, and *Trypodendron lineatum* was obtained in ethanol‐baited Lindgren Funnel traps (Table 1). For DNA extraction, the thorax and abdomen were separated using sterilized forceps and both body parts were placed into a single well of a 96‐well microtiter plate. A standard DNA extraction was carried out on each specimen following the BIOSPRINT 96 (Qiagen, Silicon Valley, CA, USA) tissue extraction protocol, by first adding 180 *μ*L ATL buffer and 20 *μ*L proteinase K to each specimen. Plates were incubated at 56°C for 10–12 h, centrifuged (500 g for 5 min), and 430 *μ*L master mix (200 *μ*L Buffer AL, 200 *μ*L Isopropanol, and 30 *μ*L MagAttract Suspension G) was added to each well. Plates were then placed in the BIOSPRINT 96 workstation and the DNA was purified following the BIOSPRINT 96 DNA Tissue program. All extracted DNA was deposited in the Frozen Collection at the Natural History Museum, London.

**Table 1:** Average number of sequences and OTUs for each beetle specimen (standard deviation in brackets) before and after rarefaction (at 1832 sequences)

Specimen	BMNH number	Number of sequences (average)	Number ofOTUs (SD)	Number of OTUs rarefied (SD)
*Hylastes ater* (specimen 1)	1047606	196,935 (101,767)	229 (40)	107 (8)
*Tomicus piniperda* (specimen 2)	1047128	6031 (3059)	54 (16)	53 (15)
*Trypodendron lineatum* (specimen 3)	1046457	28,539 (13,716)	31 (9)	28 (8)

### Tagged primer design and PCR

We used the ITS3 and ITS4 primers (White et al. 1990) to amplify the internal transcribed spacer region 2 (ITS2) (Schoch et al. 2012). The ITS2 region in fungi is 320 bp in length on average and lacks the insertions commonly found in ITS1, reducing length variation (Martin and Rygiewicz 2005; Bazzicalupo et al. 2013). These primers were modified by different 5′ identifier sequences of five to nine base pairs (Table S1) to generate 15 pairs of unique error‐correcting tags, with an edit distance of ≥3 (i.e., the minimum number of indels and substitutions that might cause misidentification of tags; Degnan and Ochman 2012), as recommended by Qiu et al. (2003). Tags were length variable to avoid problems arising from low‐diversity sequences with Illumina sequencing protocols (Gloor et al. 2010). A 2‐bp sequence was placed between the 5′ end of the primers and each tag to ensure a mismatch between the barcode and the template DNA (Zhou et al. 2011). Full‐length tagged primers were checked for hairpin formation, self‐dimerization, and cross‐dimerization using EDITTAG (Faircloth and Glenn 2012).

PCR was conducted for the three specimens with each of the 15 newly designed primer pairs to test for variability in amplification success. Each specimen–tag combination was performed in three replicates, bringing the total to 45 distinct samples per specimen. A negative control was created by performing the DNA extraction procedure on double distilled water, after which the extract was subjected to PCR with each of the 15 primer pairs. This brings the total number of amplifications to 150. For each of these, three PCRs were conducted at different annealing temperatures (50, 53, 56°C), before they were combined for sequencing. Combining multiple PCR products is a commonly used approach in microbial studies, as using a range of annealing temperatures reduces primer binding bias and counteracts the stochasticity of individual PCRs (Schmidt et al. 2013).

The following cycling conditions were used for PCRs: initial denaturation at 94°C for 300 sec, followed by 30 cycles of denaturation at 94°C for 60 sec, annealing at 50/53/56°C for 60 sec, extension at 72°C for 30 sec, and a final extension at 72°C for 120 sec. The PCR mix contained 1 *μ*L template DNA, 0.8 mmol/L dNTPs, 5 U/*μ*mol/L BIOTAQ™ DNA polymerase, 4 mmol/L MgCl2, and 0.5 *μ*mol/L of forward and reverse primers in a 40‐*μ*L reaction volume. PCR products were visualized using GelRed^™^ (Biotium, Hayward, CA, USA) on a 1% agarose gel. Purification of PCR products was performed using DNA Clean & Concentrator^™^ (Zymo Research, Irvine, CA, USA). To determine the concentration of double‐stranded DNA present, cleaned PCR products were then quantified using a Qubit 2.0 Fluorometer (Invitrogen) with the Qubit dsDNA HS Assay Kit (Invitrogen, Carlsbad, CA, USA).

After amplification, samples were pooled into 10 separate libraries following a scheme by which each replicate (set of 3) PCR is placed in a different pool (Table S2). KAPA library kits (SKU: KK8201; Kapa Biosystems, Wilmington, MA, USA) were used to prepare samples for Illumina sequencing. The library preparation followed the manufacturer's instruction, except that a step to isolate the large DNA fragments was omitted. To increase the diversity of the sequences and prevent problems arising from the low diversity of amplicons, samples were split across two separate runs and 50% of each run contained genomic DNA from an unrelated study, but the total sequencing effort equated to one full run. Paired‐end sequencing was conducted on an Illumina MiSeq sequencer with v.3 chemistry and 2 × 300 cycles.

### Bioinformatic processing

Reads from each library were quality checked with Fastqc (http://www.bioinformatics.babraham.ac.uk/projects/fastqc/,v0.11.2), and PRINSEQ‐lite 0.20.4 (Schmieder and Edwards 2011) was used to remove reads with an average Q score of <25 and to trim bases below Q25 from 3′ ends, no N's were allowed, and only read lengths of over >150 bp were retained. Fastq‐join (Aronesty 2011) was then used to merge forward and reverse reads if they matched over >50 bp at a minimum similarity of 99%.

Merged reads were renamed to mark the library from which they were derived and all libraries were then pooled together into a single Fastq file. A custom Python script (remove_multiprimer.py) (Balint et al. 2014) was used to remove primer artefacts from all reads. Sequences were demultiplexed based on the unique tags and library labels using Qiime's split_libraries.py script allowing for one‐base mismatches (Caporaso et al. 2010). Paired‐end sequences with implausible forward and reverse primer combinations were discarded. Finally, sequences were passed through the ITSx software (Bengtsson‐Palme et al. 2013), which matches input sequences to profile hidden Markov models (HMMs) (Eddy 1998) that represent kingdom‐wide sequence alignments of fungal ITS. The program removes the ITS flanking regions and also removes nonfungal sequences.

OTU clustering of ITS2 sequences was carried out with UPARSE (Edgar et al. 2011), which uses maximum parsimony models to compare each sequence to an existing OTU database. This method has been used successfully by a number of studies to analyze fungal communities (Adams et al. 2013; Balint et al. 2014; Liu et al. 2015). Briefly, the UPARSE pipeline entails the removal of singletons, that is, completely unique sequences (Brown et al. 2015), followed by dereplication to retain a single representative of each ITS2 sequence, followed by clustering. The program outputs a *fasta* file containing a single representative for each OTU. These were then checked for chimeras with UCHIME (Edgar et al. 2011). OTUs were defined by a similarity cut‐off of 97%. Finally, to check that the OTUs were truly fungal in origin, a taxonomic approach was utilized. BLASTn searches (BLAST 2.2.28+; (Altschul et al. 1990) of OTU representative sequences were conducted against the NCBI nucleotide database, and identities of each OTU representative were established with the lowest common ancestor (LCA) method in MEGAN (Huson et al. 2011). OTUs identified as nonfungal were excluded from the analysis.

### Data preparation and statistical analysis

An OTU abundance table (beetle individual × fungal OTUs) was produced with UCHIME, whereby all ITS2 sequences are mapped back against the OTU representatives at a similarity of 99% and sequence counts are used as a measure of OTU abundance. Only OTUs supported by >10 sequences were retained. OTU accumulation curves were generated for replicates of each specimen to assess whether the sequencing effort was sufficient to capture the full fungal community. This was carried out using the ‘rarecurve’ function in the R package ‘vegan’ (Oksanen et al. 2010). The saturation of curves was assessed using the ‘estimateR’ function (Table S2).

Permutational multivariate ANOVA (PERMANOVA) was conducted to test the effect of tag identity, specimen identity, and library membership on the fungal community obtained from each replicate. PERMANOVA is a nonparametric, resemblance‐based permutation method for analyzing compositional differences in communities belonging to different groups (Anderson 2006). The PERMANOVAs used pairwise dissimilarity matrices between all communities as an input, to test whether the dissimilarities between pairs of communities from all groups are significantly different from the pairwise dissimilarities of communities from the same group.

Two different pairwise dissimilarity matrices were analyzed by PERMANOVA, one based upon sequence read abundance (used here as a proxy for OTU abundance per replicate), and one based upon presence–absence (incidence). The fungal OTU abundance table was therefore used to compute two separate compositional dissimilarity matrices, each based on a different pairwise community dissimilarity index. We used the Sørensen index, which uses the incidence data, and the Bray–Curtis index, which uses abundance data.

## Results

### Read processing and OTU delimitation

**Figure 1:**
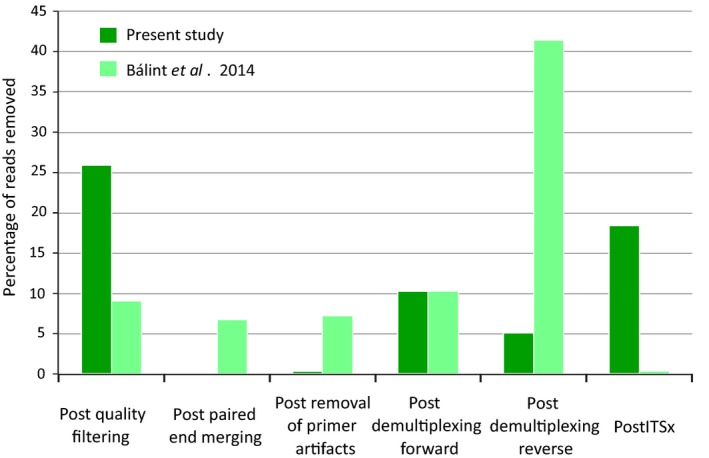
The proportion of reads removed by the bioinformatics pipeline at each processing step for the current study and the study of Balint et al. (2014).

Over 27 million paired‐end reads were obtained from one full Illumina MiSeq run. A total of 16.6 million (60.8%) reads were removed at various stages during the bioinformatics processing, leaving 10.7 million for further analysis (Fig. 1). The greatest proportion of reads (25.9% across all libraries) was lost during quality filtering and again in the final filtering step against the fungal ITS database using ITSx (18.9%) (Figs. 1, S1). Primer artefacts and complications with merging of paired reads were minimal, while unexpected combinations of forward and reverse primer tags also resulted in substantial losses of reads. The high proportion of nonfungal ITS sequences without match to the reference Hidden Markov model was evident also from the Lowest Common Ancestor (LCA) analysis (Huson et al. 2011) that included numerous sequences not assignable to any taxon, or assigned to plants and beetles (specifically Cucujiformia, consistent with the bark beetle ‘hosts’), which presumably were coamplified by the fungus‐specific primers. These sequences were no longer seen after the ITSx filtering step, except for a single OTU that was classified as nonfungal in the LCA analysis and removed.

The number of postfiltering sequences differed greatly among specimens, with *H. ater (*specimen 1) showing the highest numbers, followed by *Tr. lineatum* (specimen 3) and *To. piniperda* (specimen 2) (Table 1). Sequence numbers for each of the three specimens were largely uniform within a sample (ANOVA: *F*_(2,131)_ = 137.5; *P* < 0.0001; Table S2), indicating high reproducibility of the PCR within replicates of the same beetle DNA extract.

**Figure 2:**
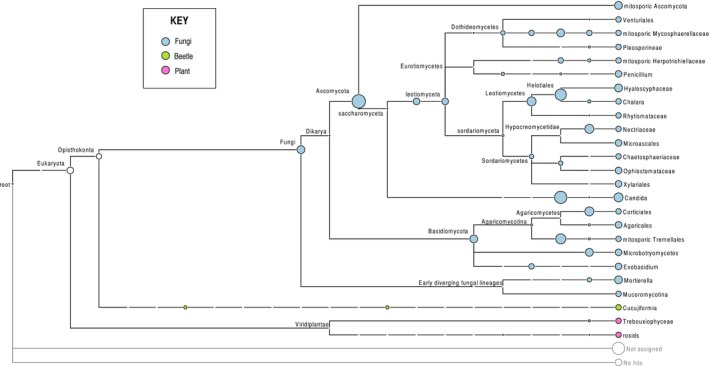
Lowest common ancestor analysis using MEGAN before ITSx treatment.

**Figure 3:**
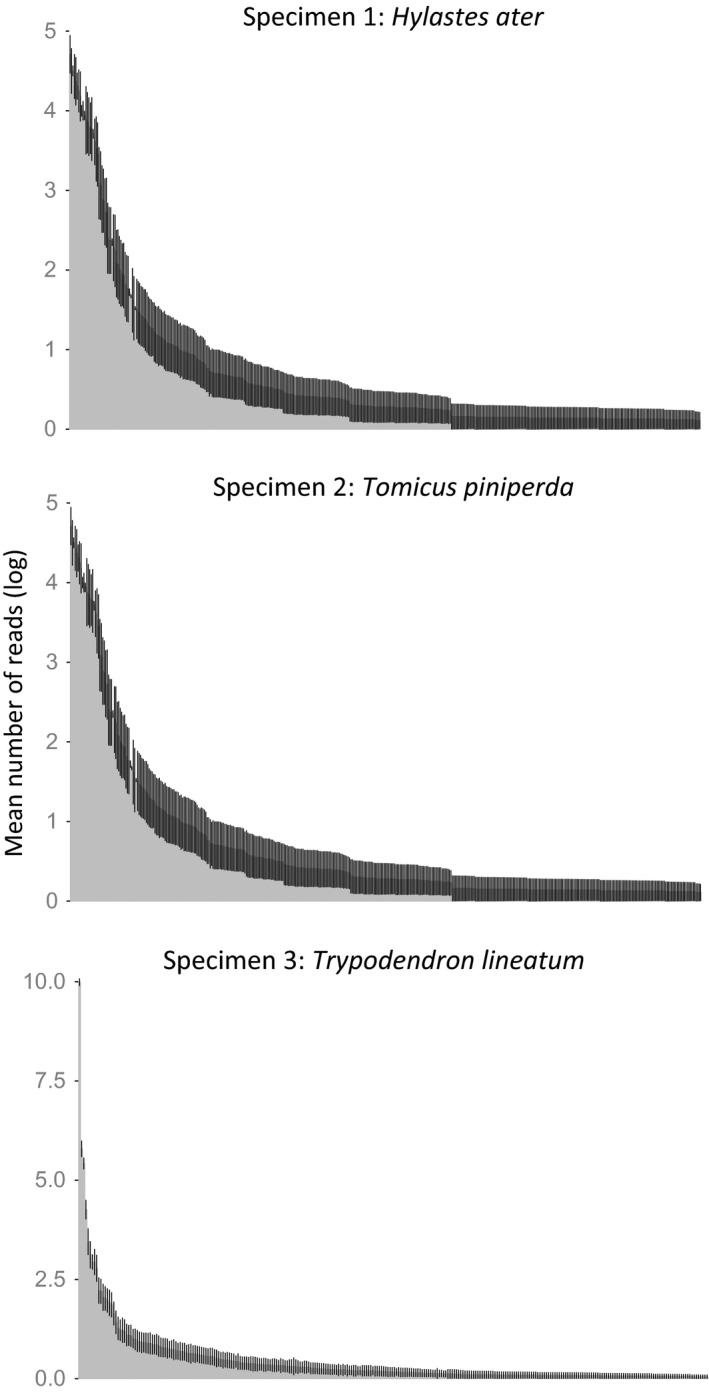
Rank abundance curve for the number of fungal OTUs showing the mean log number of reads associated with each OTU, separately for each of the three specimens used in this study. Error bars represent standard error of the mean (SEM) for each of 45 replicates (15 separate tags and three PCR replicates each).

The assembled sequences were grouped into a total of 1760 OTUs using the UPARSE software, of which 612 OTUs were removed because they were supported by fewer than 10 ITS2 sequences. Nineteen OTUs were also present in the negative control and were removed from all other samples where they occurred, and one OTU was removed because its closest Blast match was to an angiosperm (*Theobroma cacao*). This left 1128 OTUs for statistical analysis. The number of OTUs for the three specimens differed greatly, ranging from 229 OTUs on average (range = 149–323; SD = 40) for *H. ater*, 54 OTUs (range = 24–87; SD = 16) for *To. piniperda*, and 31 (range = 9–55; SD = 9) OTUs for *Tr. lineatum*. Thus, variation in OTU richness was highly correlated with the beetle specimen (ANOVA of log OTU: *F*_(2,131)_ = 589.6; *P* < 0.0001), and this was independent of the number of ITS2 sequences obtained for these specimens (Table 1). The OTUs could be assigned taxonomically to various groups of fungi including major lineages of Ascomycota and Basidiomycota using the LCA method (Fig. 2). This also includes numerous sequences placed into the classes Leotiomycetes and Sordariomycetes that include many of the tree pathogenic species, including blue stain fungi. In total, in all PCR replicates combined, there were 727 OTUs found in *H. ater* (specimen 1), 435 OTUs in *To. piniperda* (specimen 2), and 294 OTUs in *Tr. lineatum* (specimen 3). OTUs differed greatly in their abundance (number of sequences associated with an OTU), following a typical ‘hollow‐curve’ distribution of species abundance, with a few highly abundant entities and a long tail of rare ones. This abundance profile was highly reproducible among the 45 PCR replicates based on the SEM on read counts for the individual OTUs (Fig. 3).

### Reproducibility of PCR

**Figure 4:**
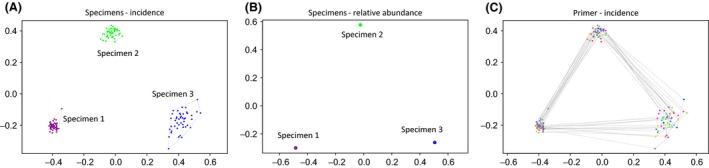
NMDS plots showing each point as the fungal community of an individual replicate based on (A and C) Sørensen (incidence) pairwise dissimilarities, (B) Bray–Curtis (abundance) pairwise dissimilarities. For (A) and (B) colors represent specimen of origin, gray polygons showing specimen groupings. For (C) colors represent the primer tag used and gray polygons show primer groupings.

A PERMANOVA analysis was conducted on the measures of dissimilarity of the communities based on incidence (presence/absence) and relative abundance of OTUs. In both tests, the sets of OTUs derived from the PCR replicates carried out on a single DNA extraction were highly similar to each other, indicating high reproducibility of the PCR in the replicates, but differed greatly from the OTU set obtained from another specimen. Specimen identity explained a high proportion of the data for OTU incidence (PERMANOVA: PseudoF = 84.21, *R* ^2^ = 0.56, *P *= 0.001), and this proportion is even higher when taking into account the OTU abundance (PseudoF = 1521.50, *R* ^2^ = 0.96, *P *= 0.001) (Fig. 4). The latter showed that the OTU set obtained from a given specimen is virtually identical for each PCR replicate (Fig. 4B).

PERMANOVAs testing the effect of primer identity on the fungal communities revealed that the primer tag used for each sample had no significant effect (PseudoF = 0.49, *R* ^2^ = 0.05, *P* = 1; Fig. 3C). The effect of library was also found to be nonsignificant (PseudoF = 1.27, *R* ^2^ = 0.01, *P *= 0.235). Thus, the choice of tag has only minimal influence on the recovered OTU set, while the library preparation was highly reproducible.

### Completeness of OTU sequencing

**Figure 5:**
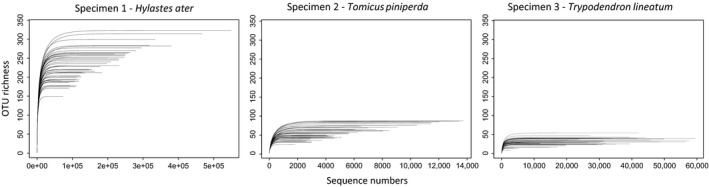
OTU accumulation curves for each of 45 PCR replicates in three specimens. Note the different scale of the horizontal axis in the three panels.

Species accumulation curves conducted on OTUs with >10 reads were fully saturated at the sequencing depth used, even for the most diverse specimen one (Fig. 5), indicating that diversity estimates would not increase with deeper sequencing. However, the replicates differed substantially in the total number of OTUs produced, indicating that PCR success of each replicate was highly variable. To account for the great variation in read numbers among replicates for comparisons of species richness in the replicates, we resampled the reads according to the lowest number of sequences of any replicate of 1832 reads. Accumulation curves of the resampled reads failed to reach the full set of OTUs in two of three cases (Fig. S2). Rarefaction based on that read number found that OTU richness was significantly reduced for specimen one (*t* = 26.40, df = 58.84, *P *< 0.0001), but not for specimen two or three (*t* = 0.16, df = 85.88, *P *< 0.87; *t* = 1.31, df = 87.12, *P *< 0.19, respectively) (Fig. S3).

## Discussion

### Reproducibility of community composition in metabarcoding

Metabarcoding is changing the way ecological communities are studied (Yu et al. 2012). The current study shows that metabarcoding of fungal communities associated with bark beetles can be performed reliably straight from DNA extractions performed on the beetle specimen without prior dissection of fungus‐bearing structures. Metabarcoding conducted in this way detected a much greater species richness than recognized previously with culturing (Giordano et al. 2012; Kolarik and Jankowiak 2013) and DNA sequencing from mycangia (Kostovcik et al. 2015). However, the methodology is dependent on the accuracy of the PCR that potentially introduces biases in the representation of species and their abundances in a natural community. Different primer tags may add to this bias and raise the question about the reproducibility and comparability of samples using this approach. Variability of PCR amplifications is not usually tested, and it is often assumed that pooling of a few PCR replicates at different annealing temperatures is sufficient to overcome the stochastic variability of the amplification process (e.g., Schmidt et al. 2013). We find that the 45 PCRs from a given sample specimen produce fungal communities that, based on pairwise community similarity, differ very little in species composition and abundance (Fig. 3). There is also a limited effect of the choice of tag sequence on the outcome of the inferred community composition. In addition, we find that the fungal OTUs are recovered in similar abundance in the replicates (Fig. 3), further demonstrating the reproducibility of the PCRs.

Despite the apparent consistency among replicates in the NMDS analysis and rank abundance curves, there was a surprising level of variation in the total number of OTUs detected among the PCR replicates (see above, and Table S3), which is also evident in the species accumulation curves that consistently reach an asymptote for each PCR replicate, but at different levels (Fig. 5; Table S3). As a proportion of the average number of species detected, the effect was strongest in the beetle specimen with the lowest number of fungal species (specimen three, *Tr. lineatum*). This type of variation is apparently not captured by the NMDS on pairwise community similarity, which would lead to the expectation that species numbers are also largely uniform. However, as this is not the case, the variation is probably affecting the rare species (tail of the hollow‐curve distribution). Detailed investigation revealed that the number of sequences supporting high‐abundance OTUs is consistent among replicates, indicating that the method is able to reliably find a core of high‐abundance OTUs, but that low‐abundance OTUs tend to be patchily distributed and inconsistent between PCR replicates (data not shown). It appears that these low‐abundance OTUs are driving the variation in OTU incidence, but that their influence is down weighted by the relative abundance analysis. Thus, the mycobiome of specimen 3 (*Tr. lineatum*) displayed the lowest diversity and the greatest variability in community composition. Conversely, specimen 1 (*H. ater*) had the highest diversity and showed the lowest variability in OTU numbers.

The observation of higher variation in OTU recovery for relatively small communities and rare species may indicate stochastic effects of the PCR amplification from mixed species communities and the chance amplification of different fungal templates if they are present in low abundance. Similar evidence is accumulating for the idea that the concentration of the original DNA extract is primarily responsible for apparently stochastic variability in microbial communities (Kennedy et al. 2014; Salter et al. 2014). Our findings are consistent with this suggestion, as low fungal diversity is likely to translate to lower DNA concentrations. Results also suggest that the fungal OTUs shared between different specimens display significantly different read abundance, further supporting the power of these methods for reproducible and accurate determination of fungal community composition, at least for the common OTUs. A caveat is that this type of reproducibility may only apply to the PCR step, but does not necessarily reflect the true diversity of the natural community whose amplification may be skewed in a systematic way (Elbrecht and Leese 2015). These PCR‐induced biases are likely to be smoothed out by conducting multiple replicates that each contribute various components of the community (Fig. 5), although this was not specifically tested here, as we did not sequence each temperature replicate separately. However, mixtures of PCR temperature variants should be used if a study aims to produce the most complete account of species representation.

### Data filtering and effect on species composition

Prior to the community analysis, extensive data filtering is needed, which potentially causes a skew in species composition and specifically may affect rare species. The initial bioinformatics data treatment resulted in removal of totally ~61% of reads. Following widely used practice, as a precaution we also removed all OTUs represented by <10 sequences, thus removing several hundred OTUs, and inevitably bias against the detection of rare species. For the bioinformatics protocols, we essentially followed the steps of the study by Balint et al. (2014) of soil fungi, albeit using different software. This approach removed an overall similar proportion of sequence reads, although they were lost at slightly different stages of the filtering process (Fig. S1). Specifically, in the current study, more data were removed in the first step of quality filtering that eliminates reads with ambiguous base calls based on Phred quality scores. This error rate depends on the specific Illumina run and possibly the quality of the template DNA used for the initial PCR step (in this case obtained from specimens in ethanol‐filled traps emptied only once per week). Our procedure was more resilient at the stage of pairing of reads and primer base calls. This step was carried out with the Fastq‐join software that uses error probabilities to accept the merged paired‐end reads, and produced hardly any incorrect combinations. However, the subsequent step of demultiplexing removed a large portion of reads, as implausible combinations of forward and reverse tags are removed, but the loss is lower than in the study by Balint et al. (2014). This high proportion of mismatched paired reads demonstrates the need for unique tagging of primers on both ends of the amplicon, as used in our approach, rather than reliance on the paired‐end tag combinations, to establish incidences of tag switching and chimera formation that would confound the subsequent OTU formation.

Finally, many sequences were removed at the ITSx stage that eliminates nonfungal sequences, which was much higher when compared with the study of Balint et al. (2014) (Fig. S1). To a small extent, this was due to coamplification from the beetle DNA templates, present in high concentrations in the DNA extracts. The LCA analysis confirmed the coamplification of Coleoptera (Fig. 2), but showed this to be a minor component besides sequences assigned to angiosperms and other unidentified sequences, and the ITSx procedure cleanly removed these. We conclude that coamplification of the beetle ‘host’ with the ITS3/4 primers is a manageable problem, while the primers pick up a great phylogenetic diversity of fungi, including the main lineages of Ascomycota and Basidiomycota. All current universal primer pairs for the amplification of fungal ITS exhibit some form of bias (Schoch et al. 2012) and those with lower levels of bias are prone to the amplification of nonfungal ITS as those from plants (Martin and Rygiewicz 2005). Standard ITS primers have been shown to exhibit taxonomic preferences (Tedersoo et al. 2014; Elbrecht and Leese 2015). Thus, a trade‐off between primer‐specificity and primer‐bias toward particular groups of fungi seems unavoidable and, if used for amplification of insect‐associated fungi, affects any alternative to the ITS3/4. Other recently designed universal primers have not yet been tested for metabarcoding, but in particular a promising EF‐1alpha primer set, showing broad amplification across most lineages of fungi (Stielow et al. 2015), may be applied in addition to the ITS. It can be expected that the detectable species composition will shift with the change to a different marker. Amplification also would need to be tested for their sensitivity to insects.

### Future improvements for metabarcoding of fungal communities

The ITS2 locus used here comprises only around 300 bp, which limits the power of this locus for species delimitation and phylogenetic placement, together with ITS length variation among lineages and intragenomic variation due to the tandem‐repeat genomic structure of the rRNA locus (Hillis and Davis 1988; Kiss 2012). In addition, concerns have been raised over the biological significance of entities defined by a single marker and simplistic cut‐off values for defining OTUs (Yamamoto and Bibby 2014). Ultimately, validated reference sequences are required for a secure assignment to taxonomic groups, using macroscopic morphological characters and culturing, where possible, to link these sequences to species and clades and to their characters. The common practice of culturing specimens from insects and placement into phylogenetic trees obtained for the ITS region (e.g., Romon et al. 2014) already constitutes a useful framework for assessing the taxonomic diversity. The ITS marker exploits highly conserved primer recognition sequences for amplification, which reduces taxonomic bias, while the intervening central region is sufficiently variable for species‐level diagnostics.

Despite the conservation of primer sites, we cannot exclude that the set of species detected with the ITS2 marker may be biased due to the variable PCR efficiency. This may be of particular relevance for the amplification of Ophiostomaceae, which include important tree pathogens and reputedly are not well represented in ITS‐based studies (Seifert et al. 2013). The LCA analysis did reveal several species of the classes Ophiostomaceae, including several OTUs grouped with *Ophiostoma*, and Leotiomycetes, including the agent of ash dieback *Chalara*. If the interest was the analyses of the Ophiostomaceae or any other specific group, the metabarcoding procedure could be adjusted to target these groups specifically with modified ITS primers or amplification of other loci. The DNA extraction method should also be considered carefully, as spores may be more resilient to efficient DNA recovery than the bark beetle tissue for which the method used in the current study was designed. Mock communities composed of defined strains could be used to test the performance of DNA extractions and amplification with particular primers. However, PCR results from such an approach may be difficult to interpret, as individual species suffer from PCR competition dependent on the precise constellation of species in the fungal communities, which cannot be replicated by contrived mixtures. Finally, the link to an external taxonomic identification is not strictly necessary if applied to a given set of samples for comparisons within a study seeking to establish internal pattern within a sample set. The ITS2 region has been the marker of choice for analogous studies of complex fungal communities of the soil and has been successful in discovering a huge uncharacterized diversity of fungi and to infer macroecological patterns (Tedersoo et al. 2014, 2015).

Sequencing straight from insect samples provides a rapid way of quantifying species richness and assessing large‐scale patterns of diversity for fungi occurring together with wood‐associated insects. Here, we establish the general principle and the required methods for the PCR and bioinformatics steps. The possibility of assessing entire fungal communities including the epi‐ and endomycetes (outer surfaces and internal parts of the insect, respectively) also increases the possibility of studying the associations of (xylo)mycetophagous bark beetle species (ambrosia beetles) that feed on the fungi and transport spores by passing them through their gut, rather than being attached to the beetle externally. The inclusion of *Tr. lineatum* in the current study presents an example of this ecological group, which here showed the lowest fungal diversity of the three specimens investigated. The design of the current study does not permit any biological conclusions from the number of fungal species encountered, but already we demonstrate a huge diversity of putative species of fungi and great differences between samples. The diversity seen here even exceeds the species numbers obtained by the only other high‐throughput sequencing study available to date (Kostovcik et al. 2015), which used the older 454/Roche sequencing technology and thus was limited by lower sequencing depth and greater rates of read errors. The rapid inventories of largely complete assemblages associated with a particular insect specimen will establish the correlation of species composition with environmental conditions, beetle host species, host trees, biogeographic distributions and other factors that can be assessed readily by metabarcoding of existing and newly collected DNA extracts from bark beetles.

## Data Accessibility

Final OTU sequence assembly: online Supporting Information.

## Conflict of Interest

None declared.

## Supporting information


**Table S1.** Experimental design showing tagged primer set and beetle specimen for each community replicate.
**Table S2.** Tagged primer sequences showing tag, linker and original primer sequence for each.
**Table S3.** Extrapolated OTU richness of each sample, Chao1 extrapolated OTU Richness is the bias‐corrected Chao non‐parametric estimation of OTU richness (O'Hara, 2005) and ACE extrapolated OTU richness is that developed by (Chiu et al., 2014).
**Figure S1.** Percentage of initial reads remaining after each processing step for each library in this study (Library 1–10) and for pooled samples from Balint et al. (2014).
**Figure S2.** Log Fungal OTU richness differences for unrarified and rarified matrices for each specimen (log scale). Only for Specimen 1 there was a significant difference between rarefied and unrarefied treatments (*P* < 0.0001).
**Figure S3.** Rarified OTU accumulation curves for each sample demonstrating that rarefaction prevents certain samples from reaching an asymptote (a) for beetle specimen 1, (b) for beetle specimen 2, and (c) for beetle specimen 3.
