# Supplementary material for: Metabarcoding of fungal communities associated with bark beetles
Source: Ecol Evol. 2016 Feb 12;6(6):1590–600. doi: 10.1002/ece3.1925 (PMC4752364; doi:10.1002/ece3.1925)

Supplementary table _1. Experimental design showing tagged primer set and beetle specimen for each community replicate. Numbers represent the library each replicate was included within.

|  | Specimen 1 | | | Specimen 2 | | | Specimen 3 | | | Negative Control |
| --- | --- | --- | --- | --- | --- | --- | --- | --- | --- | --- |
| Primer set | **Rep1** | **Rep2** | **Rep3** | **Rep1** | **Rep2** | **Rep3** | **Rep1** | **Rep2** | **Rep3** |  |
| Primer pair 1 | 1 | 10 | 9 | 8 | 7 | 6 | 5 | 4 | 3 | 2 |
| Primer pair 2 | 2 | 1 | 10 | 9 | 8 | 7 | 6 | 5 | 4 | 3 |
| Primer pair 3 | 3 | 2 | 1 | 10 | 9 | 8 | 7 | 6 | 5 | 4 |
| Primer pair 4 | 4 | 3 | 2 | 1 | 10 | 9 | 8 | 7 | 6 | 5 |
| Primer pair 5 | 5 | 4 | 3 | 2 | 1 | 10 | 9 | 8 | 7 | 6 |
| Primer pair 6 | 6 | 5 | 4 | 3 | 2 | 1 | 10 | 9 | 8 | 7 |
| Primer pair 7 | 7 | 6 | 5 | 4 | 3 | 2 | 1 | 10 | 9 | 8 |
| Primer pair 8 | 8 | 7 | 6 | 5 | 4 | 3 | 2 | 1 | 10 | 9 |
| Primer pair 9 | 9 | 8 | 7 | 6 | 5 | 4 | 3 | 2 | 1 | 10 |
| Primer pair 10 | 10 | 9 | 8 | 7 | 6 | 5 | 4 | 3 | 2 | 1 |
| Primer pair 11 | 1 | 10 | 9 | 8 | 7 | 6 | 5 | 4 | 3 | 2 |
| Primer pair 12 | 2 | 1 | 10 | 9 | 8 | 7 | 6 | 5 | 4 | 3 |
| Primer pair 13 | 3 | 2 | 1 | 10 | 9 | 8 | 7 | 6 | 5 | 4 |
| Primer pair 14 | 4 | 3 | 2 | 1 | 10 | 9 | 8 | 7 | 6 | 5 |
| Primer pair 15 | 5 | 4 | 3 | 2 | 1 | 10 | 9 | 8 | 7 | 6 |

Supplementary table 2. Tagged primer sequences showing tag, linker and original primer sequence for each.

| **Tag** | **linker** | **Original Primer** | **Final Primer (3’ 🡺5’)** | **Primer Name** |
| --- | --- | --- | --- | --- |
| CCTAT | GT | GCATCGATGAAGAACGCAGC | CCTATGTGCATCGATGAAGAACGCAGC | ITS3_1 |
| CATGA | GT | GCATCGATGAAGAACGCAGC | CATGAGTGCATCGATGAAGAACGCAGC | ITS3_2 |
| GAAGT | GT | GCATCGATGAAGAACGCAGC | GAAGTGTGCATCGATGAAGAACGCAGC | ITS3_3 |
| CGTAGA | GT | GCATCGATGAAGAACGCAGC | CGTAGAGTGCATCGATGAAGAACGCAGC | ITS3_4 |
| CTACCT | GT | GCATCGATGAAGAACGCAGC | CTACCTGTGCATCGATGAAGAACGCAGC | ITS3_5 |
| GTCGTA | GT | GCATCGATGAAGAACGCAGC | GTCGTAGTGCATCGATGAAGAACGCAGC | ITS3_6 |
| CTGAGAA | GT | GCATCGATGAAGAACGCAGC | CTGAGAAGTGCATCGATGAAGAACGCAGC | ITS3_7 |
| CCAACTA | GT | GCATCGATGAAGAACGCAGC | CCAACTAGTGCATCGATGAAGAACGCAGC | ITS3_8 |
| CTCGTAT | GT | GCATCGATGAAGAACGCAGC | CTCGTATGTGCATCGATGAAGAACGCAGC | ITS3_9 |
| GATGGTCA | GT | GCATCGATGAAGAACGCAGC | GATGGTCAGTGCATCGATGAAGAACGCAGC | ITS3_10 |
| CGGTTACA | GT | GCATCGATGAAGAACGCAGC | CGGTTACAGTGCATCGATGAAGAACGCAGC | ITS3_11 |
| GGAACCAT | GT | GCATCGATGAAGAACGCAGC | GGAACCATGTGCATCGATGAAGAACGCAGC | ITS3_12 |
| AGGATCCAA | GT | GCATCGATGAAGAACGCAGC | AGGATCCAAGTGCATCGATGAAGAACGCAGC | ITS3_13 |
| AGTTGAACG | GT | GCATCGATGAAGAACGCAGC | AGTTGAACGGTGCATCGATGAAGAACGCAGC | ITS3_14 |
| CTCCACCAT | GT | GCATCGATGAAGAACGCAGC | CTCCACCATGTGCATCGATGAAGAACGCAGC | ITS3_15 |
| ATCTG | GA | TCCTCCGCTTATTGATATGC | ATCTGGATCCTCCGCTTATTGATATGC | ITS4_1 |
| GTAAC | GA | TCCTCCGCTTATTGATATGC | GTAACGATCCTCCGCTTATTGATATGC | ITS4_2 |
| AGACT | GA | TCCTCCGCTTATTGATATGC | AGACTGATCCTCCGCTTATTGATATGC | ITS4_3 |
| CTACCT | GA | TCCTCCGCTTATTGATATGC | CTACCTGATCCTCCGCTTATTGATATGC | ITS4_4 |
| TTGGCA | GA | TCCTCCGCTTATTGATATGC | TTGGCAGATCCTCCGCTTATTGATATGC | ITS4_5 |
| AGCTAC | GA | TCCTCCGCTTATTGATATGC | AGCTACGATCCTCCGCTTATTGATATGC | ITS4_6 |
| GATCTAC | GA | TCCTCCGCTTATTGATATGC | GATCTACGATCCTCCGCTTATTGATATGC | ITS4_7 |
| CTGAGAA | GA | TCCTCCGCTTATTGATATGC | CTGAGAAGATCCTCCGCTTATTGATATGC | ITS4_8 |
| TGGTAGA | GA | TCCTCCGCTTATTGATATGC | TGGTAGAGATCCTCCGCTTATTGATATGC | ITS4_9 |
| TGGCAGAA | GA | TCCTCCGCTTATTGATATGC | TGGCAGAAGATCCTCCGCTTATTGATATGC | ITS4_10 |
| GTCCATAC | GA | TCCTCCGCTTATTGATATGC | GTCCATACGATCCTCCGCTTATTGATATGC | ITS4_11 |
| CAGAGCTA | GA | TCCTCCGCTTATTGATATGC | CAGAGCTAGATCCTCCGCTTATTGATATGC | ITS4_12 |
| GTATACTGG | GA | TCCTCCGCTTATTGATATGC | GTATACTGGGATCCTCCGCTTATTGATATGC | ITS4_13 |
| CCTAATCGA | GA | TCCTCCGCTTATTGATATGC | CCTAATCGAGATCCTCCGCTTATTGATATGC | ITS4_14 |
| AGTTGAACG | GA | TCCTCCGCTTATTGATATGC | AGTTGAACGGATCCTCCGCTTATTGATATGC | ITS4_15 |

Supplementary Table 3. Extrapolated OTU richness of each sample, Chao1 extrapolated OTU Richness is the bias-corrected Chao non-parametric estimation of OTU richness (O’Hara, 2005) and ACE extrapolated OTU richness is that developed by (Chiu *et al.*, 2014).

| Replicate name | Observed OTU Richness | Chao1 extrapolated OTU Richness | Chao1 Standard Error | ACE extrapolated OTU Richness | ACE Standard Error |
| --- | --- | --- | --- | --- | --- |
| 10Lib10 | 342 | 342 | 0 | 342 | 9.22 |
| 10Lib8 | 374 | 374 | 0 | 374 | 9.55 |
| 10Lib9 | 390 | 390 | 0 | 390 | 9.52 |
| 11Lib1 | 383 | 383 | 0 | 383 | 9.38 |
| 11Lib10 | 344 | 344 | 0 | 344 | 9.27 |
| 11Lib9 | 373 | 373 | 0 | 373 | 9.10 |
| 12Lib1 | 391 | 391 | 0 | 391 | 8.12 |
| 12Lib10 | 395 | 395 | 0 | 395 | 8.67 |
| 12Lib2 | 378 | 378 | 0 | 378 | 8.76 |
| 13Lib1 | 354 | 354 | 0 | 354 | 8.98 |
| 13Lib2 | 314 | 314 | 0 | 314 | 8.82 |
| 13Lib3 | 295 | 295 | 0 | 295 | 8.58 |
| 14Lib2 | 333 | 333 | 0 | 333 | 8.81 |
| 14Lib3 | 330 | 330 | 0 | 330 | 8.98 |
| 14Lib4 | 365 | 365 | 0 | 365 | 9.07 |
| 15Lib3 | 338 | 338 | 0 | 338 | 9.06 |
| 15Lib4 | 410 | 410 | 0 | 410 | 9.59 |
| 15Lib5 | 351 | 351 | 0 | 351 | 9.18 |
| 1Lib1 | 393 | 393 | 0 | 393 | 9.71 |
| 1Lib10 | 352 | 352 | 0 | 352 | 9.36 |
| 1Lib9 | 369 | 369 | 0 | 369 | 8.88 |
| 2Lib1 | 381 | 381 | 0 | 381 | 9.56 |
| 2Lib10 | 300 | 300 | 0 | 300 | 8.60 |
| 2Lib2 | 343 | 343 | 0 | 343 | 9.23 |
| 3Lib1 | 364 | 364 | 0 | 364 | 9.08 |
| 3Lib2 | 339 | 339 | 0 | 339 | 9.18 |
| 3Lib3 | 321 | 321 | 0 | 321 | 8.96 |
| 4Lib2 | 315 | 315 | 0 | 315 | 8.75 |
| 4Lib3 | 345 | 345 | 0 | 345 | 9.28 |
| 4Lib4 | 388 | 388 | 0 | 388 | 9.38 |
| 5Lib3 | 322 | 322 | 0 | 322 | 8.90 |
| 5Lib4 | 362 | 362 | 0 | 362 | 9.37 |
| 5Lib5 | 311 | 311 | 0 | 311 | 8.81 |
| 6Lib4 | 380 | 380 | 0 | 380 | 9.18 |
| 6Lib5 | 353 | 353 | 0 | 353 | 9.29 |
| 6Lib6 | 394 | 394 | 0 | 394 | 9.73 |
| 7Lib5 | 317 | 317 | 0 | 317 | 8.87 |
| 7Lib6 | 346 | 346 | 0 | 346 | 9.07 |
| 7Lib7 | 381 | 381 | 0 | 381 | 9.11 |
| 8Lib6 | 374 | 374 | 0 | 374 | 9.14 |
| 8Lib7 | 419 | 419 | 0 | 419 | 9.13 |
| 8Lib8 | 353 | 353 | 0 | 353 | 9.05 |
| 9Lib7 | 370 | 370 | 0 | 370 | 8.43 |
| 9Lib8 | 340 | 340 | 0 | 340 | 9.04 |
| 9Lib9 | 340 | 340 | 0 | 340 | 8.70 |
| 10Lib5 | 110 | 110 | 0 | 110 | 5.14 |
| 10Lib6 | 118 | 118 | 0 | 118 | 5.37 |
| 10Lib7 | 133 | 133 | 0 | 133 | 5.73 |
| 11Lib6 | 111 | 111 | 0 | 111 | 5.26 |
| 11Lib7 | 126 | 126 | 0 | 126 | 5.45 |
| 11Lib8 | 100 | 100 | 0 | 100 | 4.94 |
| 12Lib7 | 131 | 131 | 0 | 131 | 5.51 |
| 12Lib8 | 116 | 116 | 0 | 116 | 5.35 |
| 12Lib9 | 101 | 101 | 0 | 101 | 4.97 |
| 13Lib10 | 90 | 90 | 0 | 90 | 4.01 |
| 13Lib8 | 111 | 111 | 0 | 111 | 4.93 |
| 13Lib9 | 115 | 115 | 0 | 115 | 5.27 |
| 14Lib1 | 121 | 121 | 0 | 121 | 5.50 |
| 14Lib10 | 104 | 104 | 0 | 104 | 4.75 |
| 14Lib9 | 123 | 123 | 0 | 123 | 5.50 |
| 15Lib1 | 133 | 133 | 0 | 133 | 5.61 |
| 15Lib10 | 117 | 117 | 0 | 117 | 5.36 |
| 15Lib2 | 105 | 105 | 0 | 105 | 5.10 |
| 1Lib6 | 120 | 120 | 0 | 120 | 5.47 |
| 1Lib7 | 131 | 131 | 0 | 131 | 5.69 |
| 1Lib8 | 115 | 115 | 0 | 115 | 5.16 |
| 2Lib7 | 114 | 114 | 0 | 114 | 5.29 |
| 2Lib8 | 98 | 98 | 0 | 98 | 4.64 |
| 2Lib9 | 134 | 134 | 0 | 134 | 5.62 |
| 3Lib10 | 109 | 109 | 0 | 109 | 4.91 |
| 3Lib8 | 102 | 102 | 0 | 102 | 4.91 |
| 3Lib9 | 101 | 101 | 0 | 101 | 5.00 |
| 4Lib1 | 123 | 123 | 0 | 123 | 5.55 |
| 4Lib10 | 112 | 112 | 0 | 112 | 5.12 |
| 5Lib1 | 116 | 116 | 0 | 116 | 5.30 |
| 5Lib10 | 111 | 111 | 0 | 111 | 4.68 |
| 5Lib2 | 117 | 117 | 0 | 117 | 5.24 |
| 6Lib1 | 114 | 114 | 0 | 114 | 5.27 |
| 6Lib2 | 99 | 99 | 0 | 99 | 4.43 |
| 6Lib3 | 97 | 97 | 0 | 97 | 4.51 |
| 7Lib2 | 90 | 90 | 0 | 90 | 4.39 |
| 7Lib3 | 101 | 101 | 0 | 101 | 4.89 |
| 7Lib4 | 92 | 92 | 0 | 92 | 4.68 |
| 8Lib3 | 116 | 116 | 0 | 116 | 5.20 |
| 8Lib4 | 117 | 117 | 0 | 117 | 5.40 |
| 8Lib5 | 106 | 106 | 0 | 106 | 4.99 |
| 9Lib4 | 111 | 111 | 0 | 111 | 5.26 |
| 9Lib5 | 90 | 90 | 0 | 90 | 4.39 |
| 9Lib6 | 103 | 103 | 0 | 103 | 4.90 |
| 10Lib2 | 41 | 41 | 0 | 41 | 2.54 |
| 10Lib3 | 43 | 43 | 0 | 43 | 3.07 |
| 10Lib4 | 46 | 46 | 0 | 46 | 3.34 |
| 11Lib3 | 31 | 31 | 0 | 31 | 2.44 |
| 11Lib4 | 56 | 56 | 0 | 56 | 3.73 |
| 11Lib5 | 29 | 29 | 0 | 29 | 2.03 |
| 12Lib4 | 62 | 62 | 0 | 62 | 3.80 |
| 12Lib5 | 38 | 38 | 0 | 38 | 2.71 |
| 12Lib6 | 39 | 39 | 0 | 39 | 2.94 |
| 13Lib5 | 34 | 34 | 0 | 34 | 2.36 |
| 13Lib6 | 26 | 26 | 0 | 26 | 2.15 |
| 13Lib7 | 32 | 32 | 0 | 32 | 2.45 |
| 14Lib6 | 51 | 51 | 0 | 51 | 3.03 |
| 14Lib7 | 32 | 32 | 0 | 32 | 2.54 |
| 14Lib8 | 43 | 43 | 0 | 43 | 3.07 |
| 15Lib7 | 52 | 52 | 0 | 52 | 3.27 |
| 15Lib8 | 26 | 26 | 0 | 26 | 1.36 |
| 15Lib9 | 37 | 37 | 0 | 37 | 2.24 |
| 1Lib3 | 58 | 58 | 0 | 58 | 3.66 |
| 1Lib4 | 53 | 53 | 0 | 53 | 3.28 |
| 1Lib5 | 42 | 42 | 0 | 42 | 2.27 |
| 2Lib4 | 66 | 66 | 0 | 66 | 3.03 |
| 2Lib5 | 40 | 40 | 0 | 40 | 2.64 |
| 2Lib6 | 53 | 53 | 0 | 53 | 3.21 |
| 3Lib5 | 60 | 60 | 0 | 60 | 3.28 |
| 3Lib6 | 41 | 41 | 0 | 41 | 2.75 |
| 3Lib7 | 48 | 48 | 0 | 48 | 3.31 |
| 4Lib6 | 61 | 61 | 0 | 61 | 3.56 |
| 4Lib7 | 46 | 46 | 0 | 46 | 3.05 |
| 4Lib8 | 25 | 25 | 0 | 25 | 1.36 |
| 5Lib7 | 57 | 57 | 0 | 57 | 3.51 |
| 5Lib8 | 32 | 32 | 0 | 32 | 2.34 |
| 5Lib9 | 34 | 34 | 0 | 34 | 1.88 |
| 6Lib10 | 44 | 44 | 0 | 44 | 2.11 |
| 6Lib8 | 56 | 56 | 0 | 56 | 3.54 |
| 6Lib9 | 43 | 43 | 0 | 43 | 2.67 |
| 7Lib1 | 14 | 14 | 0 | 14 | 1.79 |
| 7Lib10 | 31 | 31 | 0 | 31 | 2.75 |
| 7Lib9 | 33 | 33 | 0 | 33 | 2.76 |
| 8Lib1 | 60 | 60 | 0 | 60 | 3.69 |
| 8Lib10 | 44 | 44 | 0 | 44 | 2.68 |
| 8Lib2 | 36 | 36 | 0 | 36 | 2.69 |
| 9Lib1 | 55 | 55 | 0 | 55 | 3.70 |
| 9Lib2 | 42 | 42 | 0 | 42 | 3.18 |
| 9Lib3 | 22 | 22 | 0 | 22 | 1.97 |

Supplementary Fig. S1. Percentage of initial reads remaining after each processing step for each library in this study (Library 1 – 10) and for pooled samples from Bálint *et al.* (2014)

Supplementary Fig. S2. Log fungal OTU richness differences for unrarified and rarified matrices for each specimen. Only for Specimen 1 there was a significant difference between rarefied and unrarefied treatments (*p* < 0.0001).


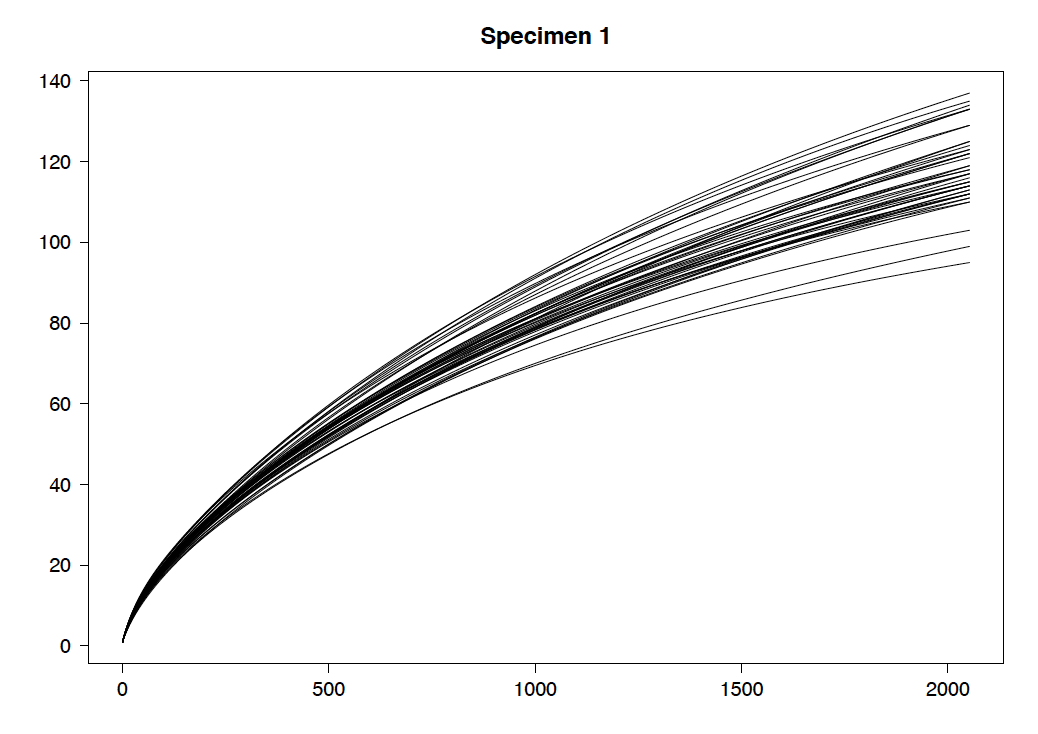

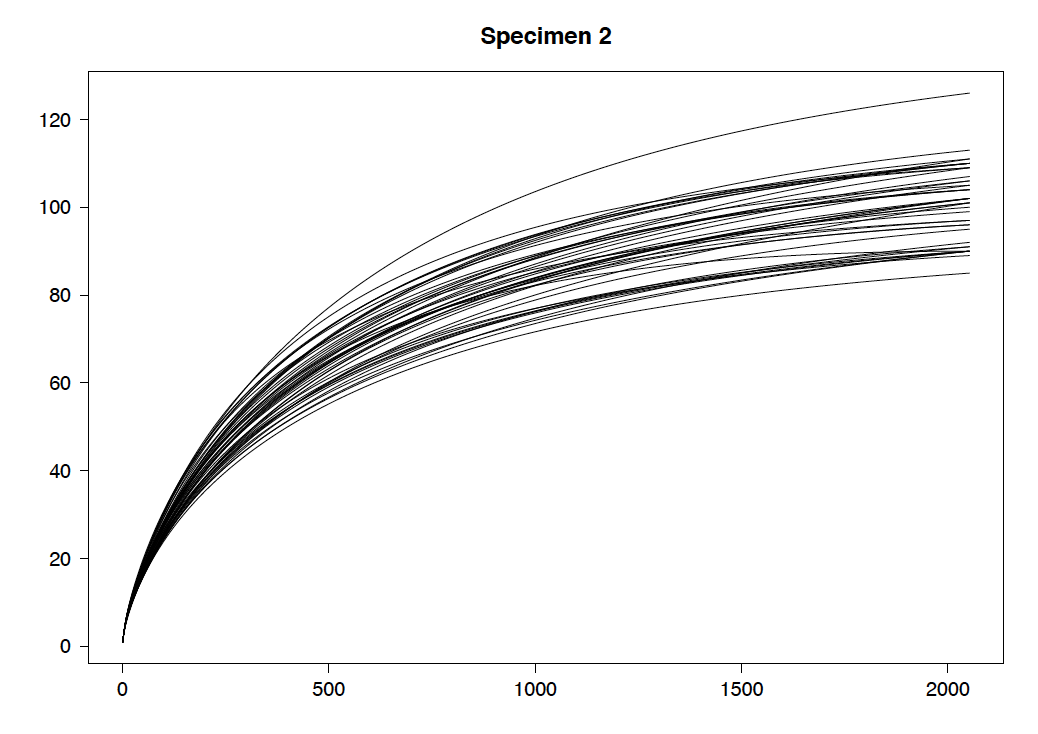

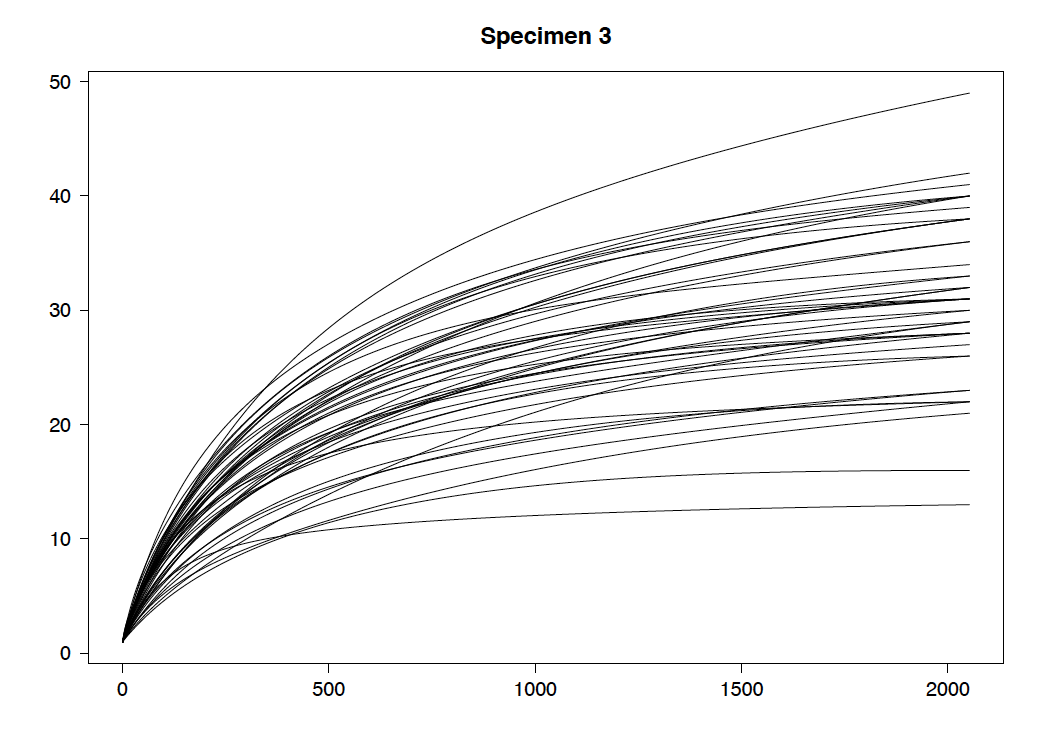


Supplementary Fig. S3. Rarified OTU accumulation curves for each sample demonstrating that rarefaction prevents certain samples from reaching an asymptote a) for beetle specimen 1, b) for beetle specimen 2 and c) for beetle specimen 3.


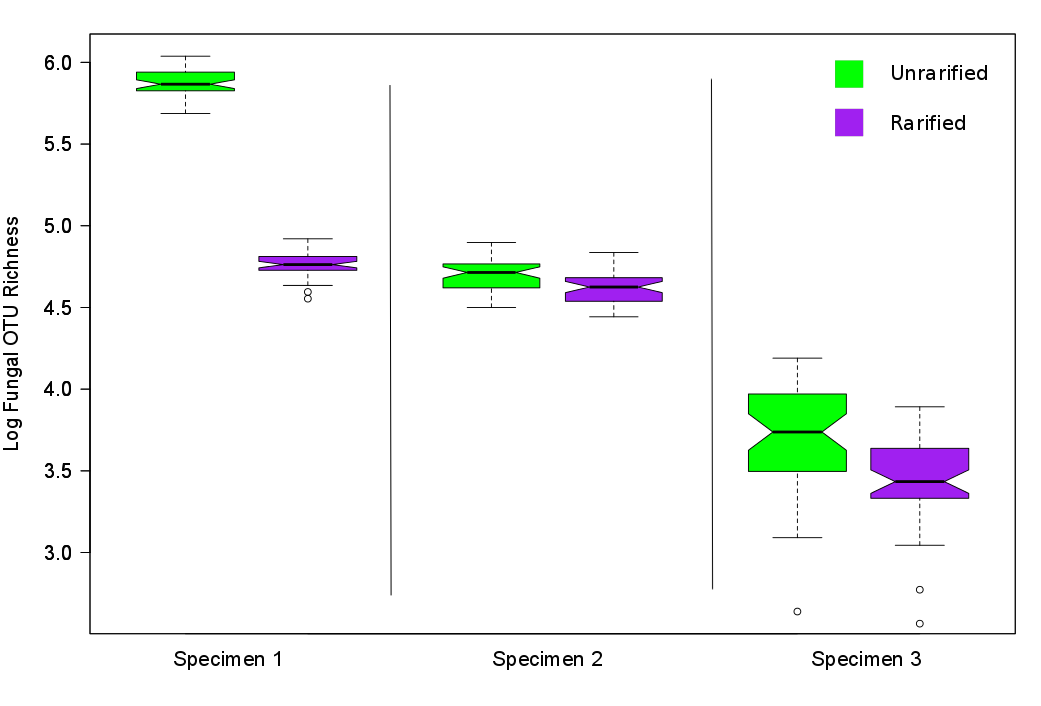

Supplement: Supplementary file 1 — Table S1. Experimental design showing tagged primer set and beetle specimen for each community replicate. Table S2. Tagged primer sequences showing tag, linker and original primer sequence for each. Table S3. Extrapolated OTU richness of each sample, Chao1 extrapolated OTU Richness is the bias‐corrected Chao non‐parametric estimation of OTU richness (O'Hara, 2005) and ACE extrapolated OTU richness is that developed by (Chiu et al., 2014). Figure S1. Percentage of initial reads remaining after each processing step for each library in this study (Library 1–10) and for pooled samples from Balint et al. (2014). Figure S2. Log Fungal OTU richness differences for unrarified and rarified matrices for each specimen (log scale). Only for Specimen 1 there was a significant difference between rarefied and unrarefied treatments (P < 0.0001). Figure S3. Rarified OTU accumulation curves for each sample demonstrating that rarefaction prevents certain samples from reaching an asymptote (a) for beetle specimen 1, (b) for beetle specimen 2, and (c) for beetle specimen 3. [file ECE3-6-1590-s001.docx]
